# Syn-Vivo Bioerosion of *Nautilus* by Endo- and Epilithic Foraminiferans (New Caledonia and Vanuatu)

**DOI:** 10.1371/journal.pone.0125558

**Published:** 2015-04-20

**Authors:** Barbara Seuss, Max Wisshak, Royal H. Mapes, Neil H. Landman

**Affiliations:** 1 Friedrich-Alexander Universität Erlangen-Nürnberg, GeoZentrum Nordbayern—Paläoumwelt, Loewenichstraße 28, 91054 Erlangen, Germany; 2 Senckenberg am Meer, Marine Research Department, Südstrand 40, 26382 Wilhelmshaven, Germany; 3 North Carolina Museum of Natural Sciences, 11 West Jones Street, Raleigh, NC 27601, United States of America; 4 American Museum of Natural History, Central Park West at 79^th^ Street, New York City, NY 10024–5192, United States of America; University of Bologna, ITALY

## Abstract

A variety of *syn-vivo* bioerosion traces produced by foraminiferans is recorded in shells of *Nautilus* sampled near New Caledonia and Vanuatu. These are two types of attachment scars of epilithic foraminiferans and two forms of previously undescribed microborings, a spiral-shaped and a dendritic one, both most likely being the work of endolithic 'naked' foraminiferans. Scanning electron microscopy of epoxy-resin casts of the latter revealed that these traces occur in clusters of up to many dozen individuals and potentially are substrate-specific. The foraminiferan traces are the sole signs of bioerosion in the studied *Nautilus* conchs, and neither traces of phototrophic nor other chemotrophic microendoliths were found. While the complete absence of photoautotrophic endoliths would be in good accordance with the life habit of *Nautilus*, which resides in aphotic deep marine environments and seeks shallower waters in the photic zone for feeding only during night-time, the absence of any microbial bioerosion may also be explained by an effective defence provided by the nautilid periostracum. Following this line of reasoning, the recorded foraminiferan bioerosion traces in turn would identify their trace makers as being specialized in their ability to penetrate the periostracum barrier and to bioerode the shell of modern *Nautilus*.

## Introduction

Recent nautilids have increasingly received attention by biologists and paleontologists [1–5] because *Nautilus* and *Allonautilus* are the only extant representatives of externally shelled cephalopods, a group of molluscs that was highly abundant and diverse throughout the Paleozoic and the Mesozoic. They are thus used as modern analogues of ammonoids for studying shell growth, function, and taphonomy [6–8]. Over the past 20 years catches of live *Nautilus* by R. H. Mapes and colleagues from various regions in the Southern Pacific Ocean (compare Figure 2 in [9]) were studied in respect of evolutionary radiation. Many of these shells were encrusted by epilithic organisms [10] and show signs of bioerosion.

The term 'bioerosion' was first defined by [11] as “the removal of consolidated mineral or lithic substrate by the direct action of organisms”, which is a major factor in the degradation of carbonate skeletal material. Bioerosion comprises chemical (dissolution) and mechanical (biting, crushing, gnawing) processes and the reasons are manifold, including grazing on epiliths, feeding on substrate components, attachment, parasitism, and most importantly protection [12]. Four main categories of bioerosive organisms include grazers, macroborers (traces >1 mm), microborers (traces <1 mm), and organisms that etch attachment scars [13–18]. Traces produced by bioeroding microendoliths are preserved as trace fossils throughout the Phanerozoic and commonly closely resemble the body shape of their producer [19]. Based on the knowledge of the environmental demands of their recent producers, the analysis of ichnocoenoses (i.e., assemblages of ichnotaxa) in fossils helps to reconstruct environmental conditions at the time that the fossil organisms lived and secreted the hard parts. Especially in the reconstruction of paleobathymetry, ichnocoenosis analysis is an approved tool, and current research is additionally directed towards reconstructing paleosalinity, paleotemperature, and paleotrophodynamics (see [18] for a review).

**Fig 1:**
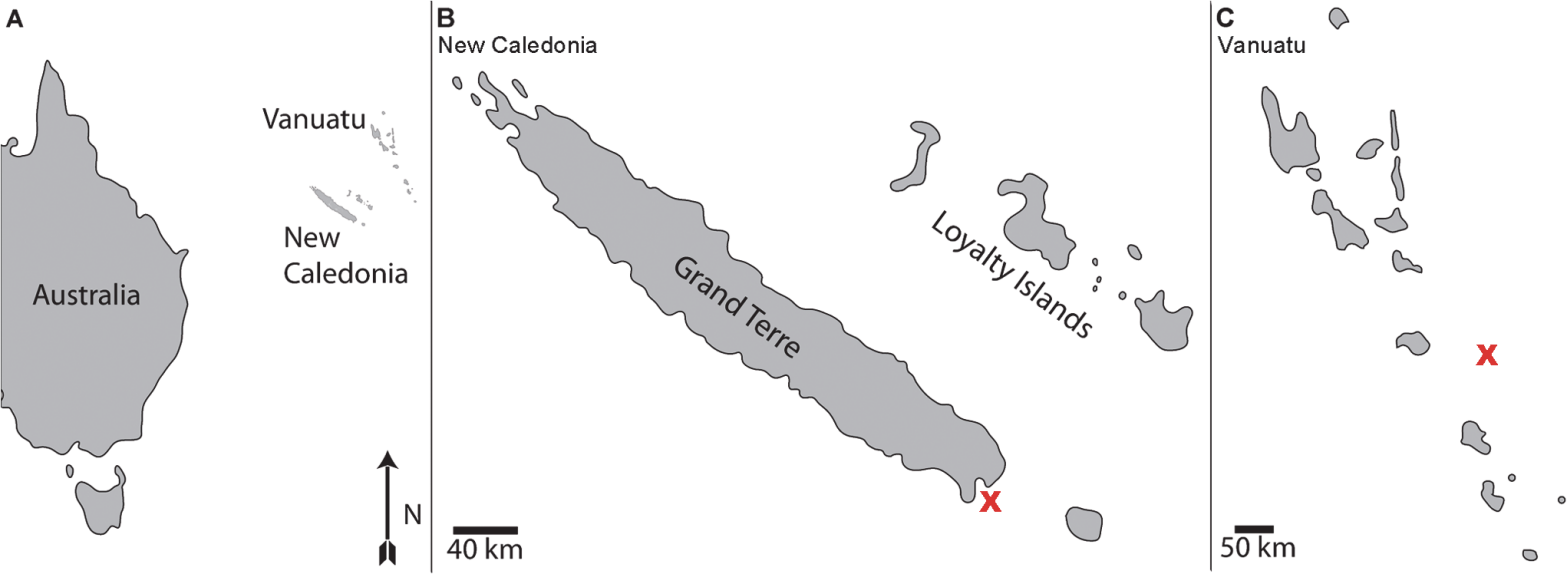
Geographical position of the sample sites in the New Caledonia and Vanuatu regions. (A) Sampling sites (New Caledonia and Vanuatu) of the *Nautilus* specimens east of Australia. (B) Sampling site of *Nautilus macromphalus* specimens near New Caledonia marked with a cross. (C) Sampling site of *Nautilus pompilius* near Vanuatu marked with a cross.

**Fig 2:**
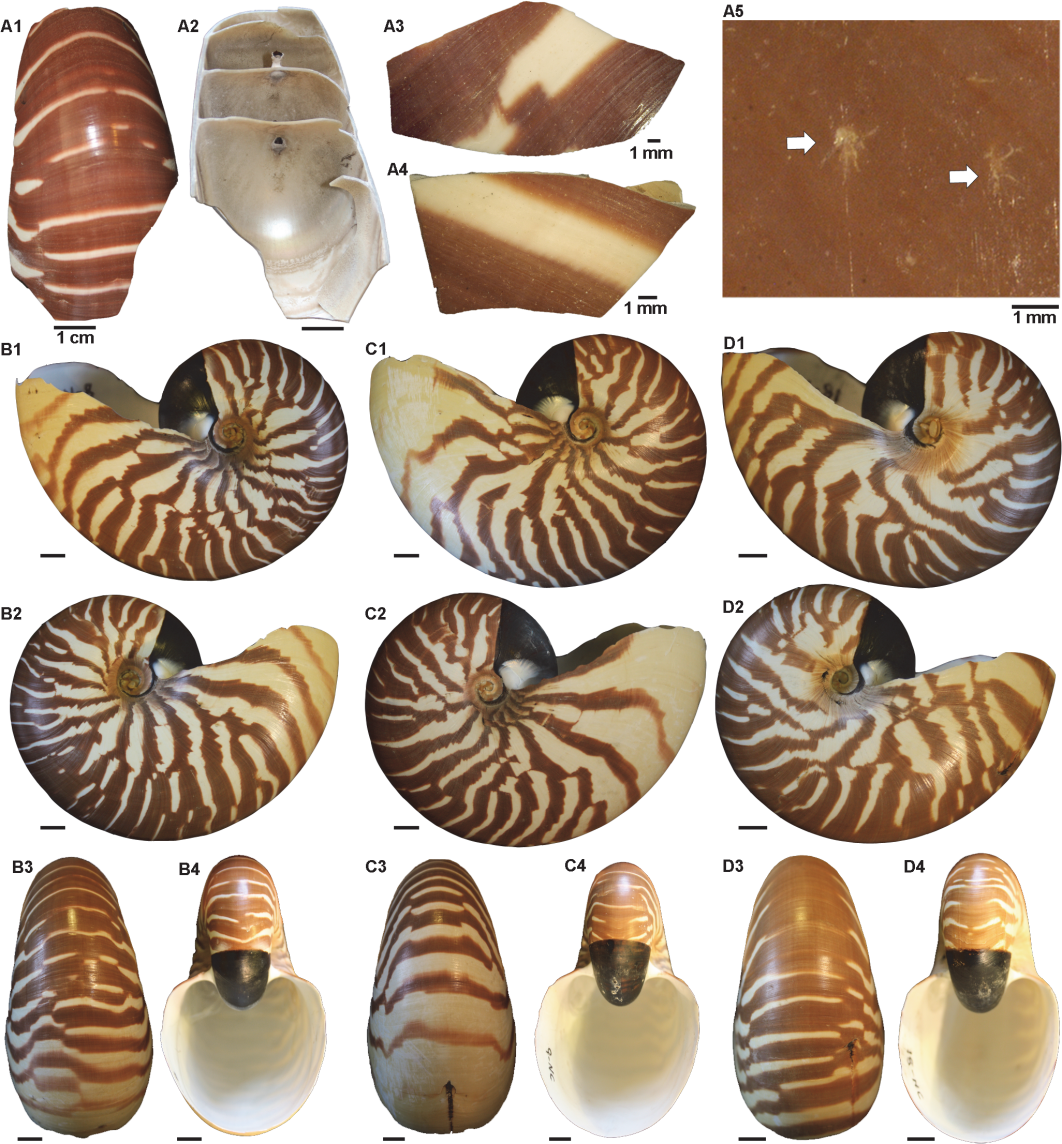
Shell fragments of the *Nautilus pompilius* specimen from Vanuatu (AMNH 310431) and the three *Nautilus macromphalus* specimens from New Caledonia. (A) *N*. *pompilius*. (A3-A4) Exemplary fragments of the shell. (A5) Dentritic structures on the shell surface produced by boring ‘naked’ foraminiferans (producing morphotype 3). (B) Specimen AMNH 93431, *N*. *macromphalus*. (C) Specimen AMNH 93432, *N*. *macromphalus*. (D) Specimen AMNH 93433, *N*. *macromphalus*. Scale bar for the shells is 1 cm unless indicated otherwise.

In the present study, we investigate previously unknown traces of *syn-vivo* bioerosion in four specimens of *Nautilus* from Vanuatu (Figs 1A, 1C and 2A) and New Caledonia (Figs 1A and 1B and 2B–2D), and discuss their environmental and taphonomical implications.

## Material and Methods

The two studied species of *Nautilus* (Table 1) include one individual of *N*. *pompilius* (collection number AMNH 310431) (Fig 2A) from Vanuatu [9] and three specimens of *N*. *macromphalus* (collection numbers AMNH 93431–93433) (Fig 2B–2D) from New Caledonia. The remains of *N*. *pompilius* comprise several fragments of a single conch (broken during shipping) while the shells of *N*. *macromphalus* are well preserved and intact.

**Table 1:** Sample specifications of the four studied *Nautilus* specimens.

Specimen (AMNH)	310431	93431	93432	93433
Species	*Nautilus pompilius*	*Nautilus macromphalus*		
Figures	2A	2B	2C	2D
Locality	Vanuatu	New Caledonia		
Latitude / Longitude	17° 46' 09'' S / 169° 09' 06'' E	22° 24' 26'' S / 167° 03' 33'' E		
Caught in	2004	2008	2002	2002
Shell diameter (in mm)	77	132	137	115
Maturity	mature	late juvenile	late juvenile	juvenile
Shell abrasion and color loss	none			
# of sub-samples	6	15	14	16
# of epoxy-resin casts	20	38	35	47

After macro- and microscopical investigation regarding shell preservation and surface structures, epoxy-resin casts were prepared following the protocol in [18] based on the cast-embedding technique invented by [20–21]. Thereby subsamples (approximately 1 x 1 cm in size) were impregnated with epoxy resin (Araldite BY 18 + Aradur 21) in a vacuum chamber (Struers Epovac / Citovac). After curing, the resin blocks were formatted and one half of each sample was used for partial etching while the other half was completely decalcified using diluted HCl. The casts, now showing the positive infill of the bioerosion traces, were rinsed with purified water, dried, mounted, and sputter-coated with gold (Cressington 108auto) for investigation with a Scanning Electron Microscope (Tescan Vega\\xmu).

### Ethics statement

In this study one specimen of *Nautilus pompilius* (Mollusca: Cephalopoda) was used. The species is not yet endangered or protected. The specimen was collected with approval of the Department of Fisheries and Environment Unit of Vanuatu. *Nautilus macromphalus* (Mollusca: Cephalopoda) is a locally protected species. Permission to collect specimens was approved by J. Fourmy, director of the Department of Environment, Province Sud, New Caledonia. Import of the specimens to the American Museum of Natural History (AMNH), New York was authorized by the U.S. Fish and Wildlife Service. Copies of the permits are available at the American Museum of Natural History where the specimens are deposited.

## Results

Four types of *syn-vivo* bioerosion traces were recorded in and on the two species of *Nautilus*, none of which could be assigned to existing ichnospecies, thus leaving them with informal nomenclature until adequate fossil holotype material becomes available.

**Fig 3:**
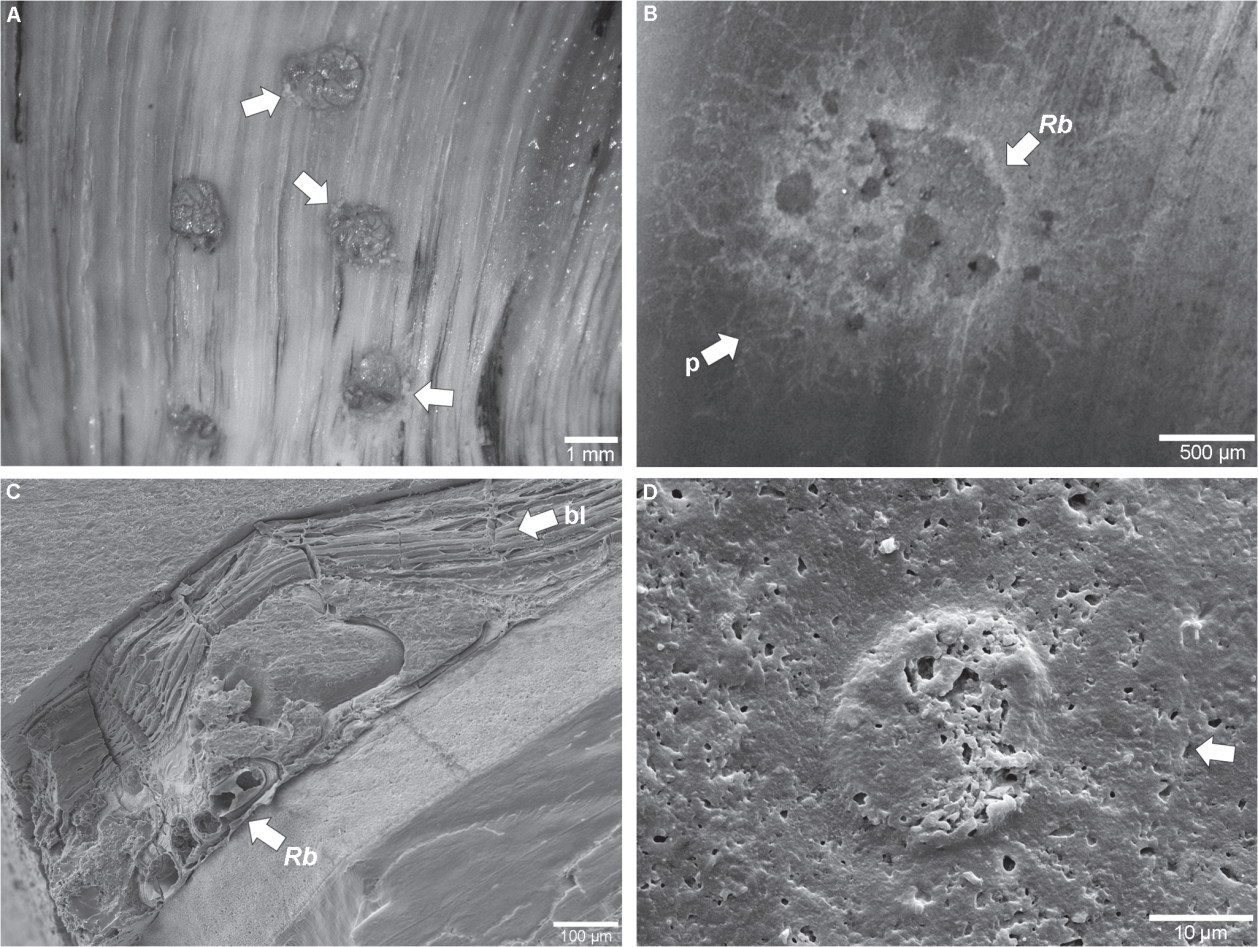
Attachment scars on the shells of *Nautilus macromphalus* from New Caledonia. (A) Cluster of *Rosalina bradyi* attached to the shell of specimen AMNH 93433. (B) Attachment scar of *R*. *bradyi* (Morphotype 1) (*Rb*) on the shell of specimen AMNH 93431; remains of the fringe of pseudopodia indicated with 'p'. (C) SEM image of *R*. *bradyi* (*Rb*) attached to the *Nautilus* shell and overgrown by the black layer (bl). (D) SEM image of epoxy resin casts showing an attachment scar (Morphotype 2) of a foraminifer specimen with affinity to *Gypsina vesicularis*; the center displays a spiral morphology that is surrounded by a circular depression (arrow) (specimen AMNH 93431).

Morphotype 1 represents surficial foraminiferan (Fig 3A) attachment scars (Fig 3B). A few dozen traces of this kind were recorded. The diameter of each shallow scar measures up to 1.5 mm and reflects the outline and spiral organisation of the foraminiferan test. Some of the traces show a distinct fringe of pseudopodia remnants (Fig 3B). Some of the *in situ* foraminiferans and their attachment scars were found overgrown by the black layer (Fig 3C) and subsequent shell growth of the nautilid’s body chamber, thereby producing distinct notches. The trace was present in the New Caledonia material only.

Morphotype 2 (Fig 3D) is an attachment scar that consists of an inner depression with a spiral-shaped organization and an outer concentric groove. The inner depression of the specimen is slightly oval and 25 μm in maximum diameter. The outer circle is 50 μm in diameter and does not display any ornamentation. Only a single trace of this type was found which was present in the New Caledonia material only.

**Fig 4:**
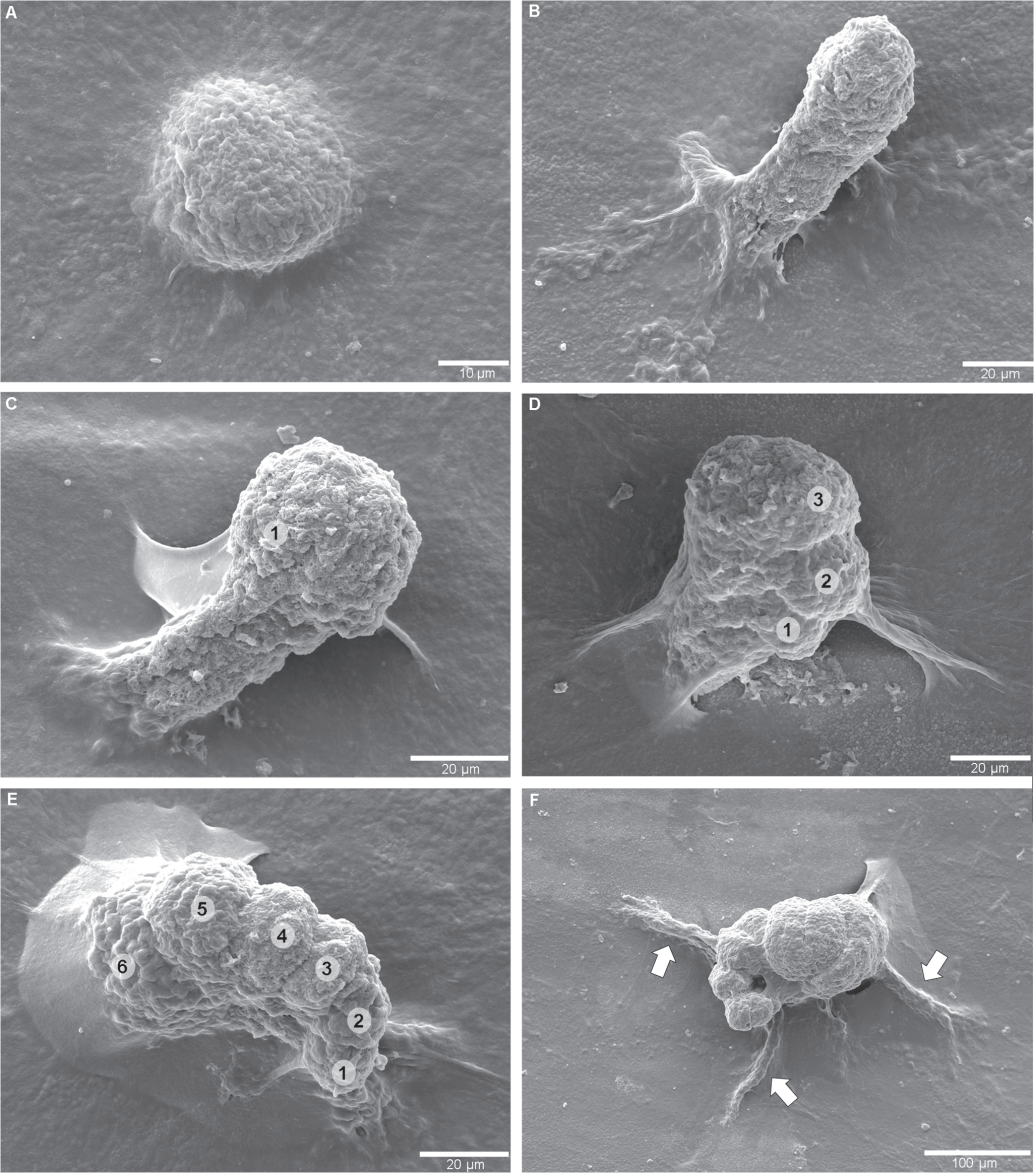
Ontogeny of Morphotype 3 in *Nautilus macromphalus* from New Caledonia as seen in SEM images of epoxy-resin casts. (A) Initial boring. (B) An initial tunnel is bored into the substrate. (C) The termination of the stalk forms the first swelling (①). (D) The trace continues to extend into the substrate and begins to form incremental lateral swellings (①–③). (E) The number of increments arranged as a spiral increases (①–⑥). (F) Mature morphology with a spiral of numerous increments, a large chamber, and connections to the substrate via radiating rhizoidal galleries (arrows).

**Fig 5:**
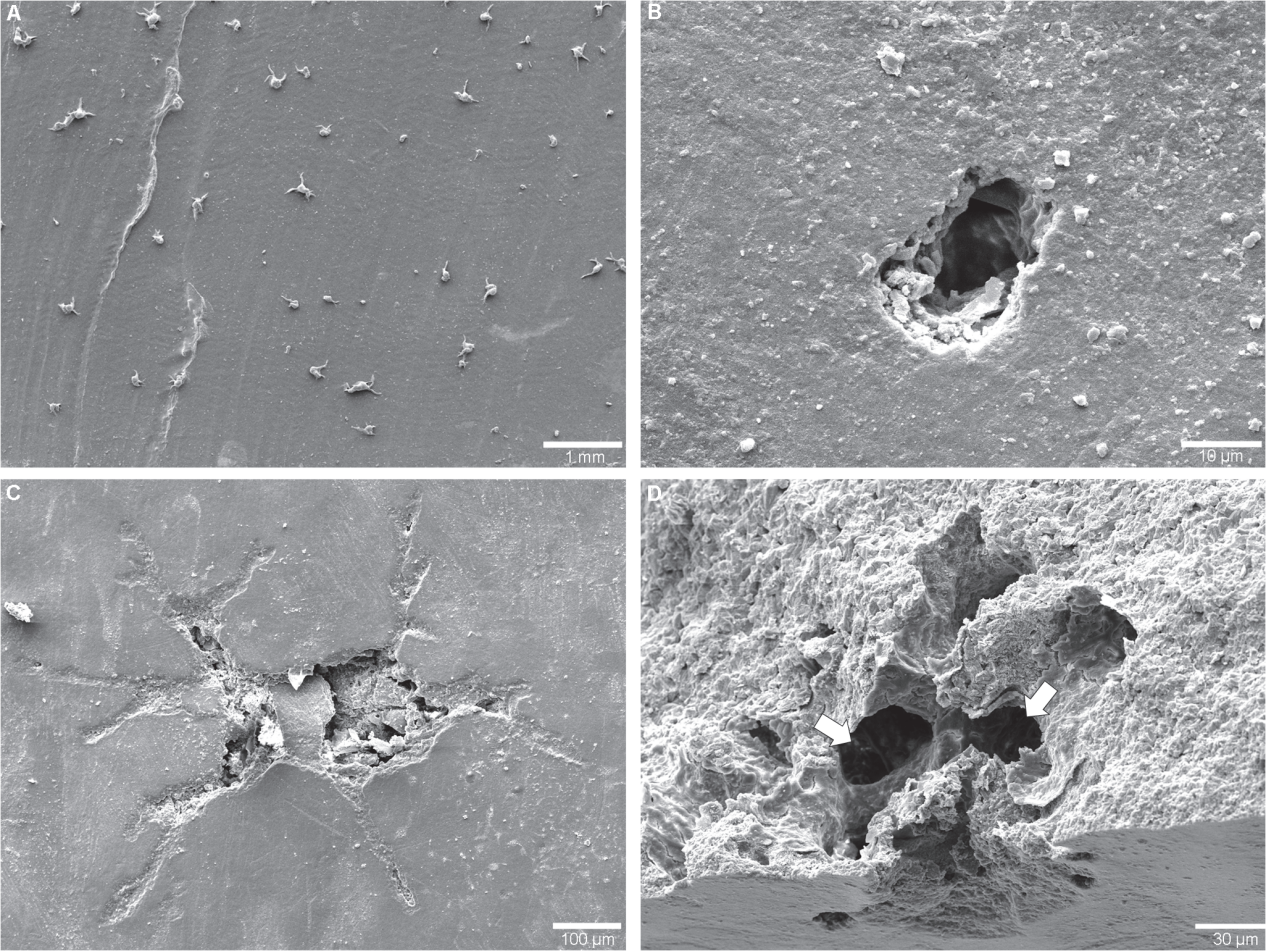
Morphotype 3 in *Nautilus macromphalus* from New Caledonia as seen in SEM images of epoxy-resin casts. (A) Cluster of traces in an epoxy-resin cast. (B) Photograph of shell surface, showing the entrance pit produced by the foraminifer. (C) Shell surface featuring the mature stage with large main chamber and pronounced rhizoids. (D) Broken shell allowing insight of the cavity produced by the 'naked' foraminifer with organic remains and two chambers (arrows).

Morphotype 3 (Figs 4 and 5) is very abundant in the synthetic resin casts of the *Nautilus* shells (a few hundred specimens) and occurs either as isolated individuals or as clusters (Fig 5A). The individual traces initiate from a single point of entry on the outer surface of the shell (Figs 4A and 5B–5D). Referring to the ontogenetic series recorded in the positive relief of the resin casts, after producing a stalk (Fig 4B), an initial swelling at the stalk's termination is formed (Fig 4C). More advanced ontogenetic stages display a spiral of incremental swellings (Fig 4D and 4E) before rhizoidal galleries evolve (Fig 4F). The latter connect the main chamber to the substrate surface. In the mature stage a large bulge with incremented spiral appendix and an extended and branching rhizoidal gallery-system (Figs 4F and 5C) is developed. The size of the central spiral in mature specimens is up to 160 μm in diameter and 150 μm in penetration depth, and the entire trace with the rhizoidal appendages reaches a maximum of 600 μm in diameter. Fig 5D illustrates entry and penetration of this morphotype into the shell of a *N*. *macromphalus* specimen. This morphotype is present in both *N*. *pompilius* from Vanuatu and *N*. *macromphalus* from New Caledonia.

**Fig 6:**
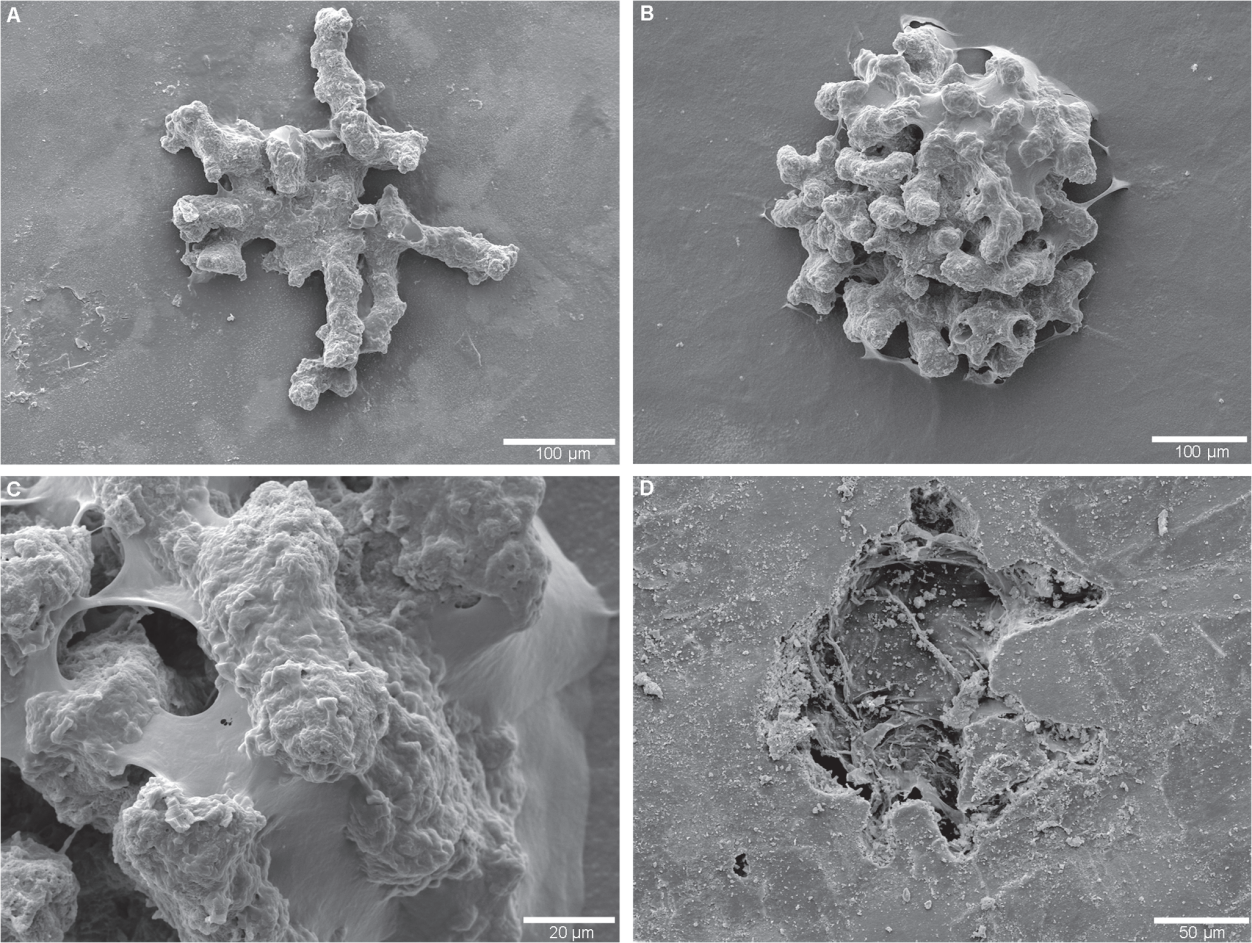
Morphotype 4 in *Nautilus macromphalus* from New Caledonia. (A) SEM image of initial borings of the foraminifer in an epoxy-resin cast. (B) Mature morphology with numerous irregularly branching galleries forming a hemispherical plexus. (C) Close-up of galleries displaying their irregular surface texture. (D) SEM-photograph of shell surface illustrating the entrance of the foraminiferan trace.

Morphotype 4 (Fig 6) is a dendritic microboring. In the casts it is less abundant than Morphotype 3, with some tens of specimens. Initial ontogenetic stages of the trace (Fig 6A) show an irregular organization of branching galleries, while mature specimens (Fig 6B) display a more regular hemispherical arrangement. Mature traces are several hundred microns in diameter and reach up to 150–200 μm in penetration depth. Individual galleries are 40–50 μm in diameter, bifurcate or anastomose (Fig 6B), and have an irregular surface texture that may exhibit short protrusions (Fig 6C). The terminations of the galleries are blunt to round. The irregularly shaped entry into the shell is illustrated in Fig 6D. This type of trace was only present in the subsamples of *N*. *macromphalus* from New Caledonia.

## Discussion

We present the first report on *syn-vivo* bioerosion in the shells of *N*. *pompilius* and *N*. *macromphalus*, in the form of scars left by epilithic foraminiferans (morphotypes 1 and 2) and microborings produced by endolithic foraminiferans (morphotypes 3 and 4).

[22] published a compilation on the foraminiferans from the New Caledonia area, none of which is known to bioerode hard substrate. However, it is well established that several species of foraminiferans do bioerode. In *Halimeda* (chlorophyte), [23] discovered different ontogenetic stages of foraminiferan cavities that closely resemble the shape of the producers and therefore excluded the idea that the foraminiferans inhabit pre-existing holes. Additionally, [24] reviewed 20 species that actively produce cavities and attachment scars in hard substrates and [25] (and references therein) extended this number to 23 known species. These detailed ichnotaxonomic studies comprise five ichnogenera, none of which resembles the morphotypes reported herein. Most occurrences of endolithic foraminiferans are known from tropical shallow-water coral reef environments like Mauritius, the Red Sea or the Coral Sea [24–25], but foraminiferans are also common in warm- and cold-temperate settings where they are abundant already in initial stages of bioerosion [17, 26]. Even though bioeroding foraminiferans appear more abundant in shallow waters, one species (*Rosalina carnivora* bioeroding the bivalve *Acesta angolensis*) was found in water depths of up to 951 m [24, 27].

All foraminiferans reported by [24] are test-bearing species, but microboring bioerosion traces are also the work of so-called 'naked' foraminiferans (no shell or soft shell) that produce dendritic microborings (ichnofamily: Dendrinidae), such as the common *Semidendrina pulchra*, as established and discussed by [28]. The fossil record of the latter ichnospecies extends back to the Carboniferous [29] and its producing foraminiferan, *Globodendrina monile*, was reported from the Callovian of southern England and northern France [30].

Bioerosion by foraminiferans is not restricted to dead substrate but was also reported from living organisms such as parasitism by foraminiferans infesting echinoids during the Cretaceous [31] or the conspicuous *Hyrrokkin sarcophaga* parasitizing on, for example, cold-water corals and large bivalves [25].

The bioerosion traces encountered on and in the present specimens of the live-caught *Nautilus* are addressed as the work of foraminiferans following several lines of reasoning:

- Morphotype 1 is assigned to the trochospiral foraminiferan *Rosalina bradyi* (pers. comm. J.-P. Debenay) because the producer of the traces was found *in situ* (Fig 3A) and the traces reflect its outline and spiral architecture.
- Morphotype 2 (Fig 3D) has closest affinity to traces produced by the epilithic foraminiferan *Gypsina vesicularis*, as reported by [17] and [26], albeit it is much smaller in size and thus was probably produced either by a juvenile specimen or a related smaller species. Its inner circular shaped pit shows spiral morphology that resembles the organization of chambers of rotaliid foraminiferans.
- Morphotype 3 (Figs 4 and 5) strongly resembles chambered foraminiferans in both morphology and ontogenetic sequence. We were not able to detect any foraminiferan tests in the shells (Fig 5B–5D) and thus follow the line of reasoning put forward for *Semidendrina pulchra* by [28] who interpret the latter trace as the work of a 'naked' foraminiferan.
- Morphotype 4 resembles the plexus of *Semidendrina* as described by [28] only that in the present case the plexus forms a hemispherical dome rather than a semi-circular plexus originating to one side from a main chamber. The irregularly branching and anastomosing character and spiny micro-texture of the individual galleries are in good accordance to *S*. *pulchra* and to the interpretation of foraminiferan pseudopodia.

Our observations and interpretations imply that all *syn-vivo* traces recorded in and on the studied shells of *Nautilus* from New Caledonia and Vanuatu are produced by foraminiferans.

No traces of photoautotrophic bioeroding organisms such as algae or cyanobacteria were found in the present material, potentially indicating an aphotic environment, which is in good accordance to the preferred habitat of *Nautilus*, living in deeper marine environments during daytime and rising in the water column for feeding during the night. Since there is also a complete lack of chemotrophic microboring fungi, which are commonly ubiquitous in aphotic ichnocoenoses (see [32] for a review), a likely explanation for the lack of microbial bioerosion is the effective protection provided by the organic outermost shell layer, the periostracum, even though it is rather thin in *Nautilus*, particularly in mature stages (this will need further testing, as only a single shell was available for this study). According to [33] the periostracum is one of the most chemically inert substances that can be produced by animals and it is also one of the mechanically strongest. However, there are various organisms that are capable of invading or penetrating the periostracum. Traces of microbial bioerosion from live cf. *Bathymodiolus* (Mollusca: Bivalvia) and experimentally exposed *Solemya* (Mollusca: Bivalvia) [34–35] and a limited number of further studies [36–38] report endoliths that are capable of destructing the periostracum and the shell during the lifetime of the organism. The ability to penetrate the periostracum might depend on the construction, thickness, and composition, as well as on specialized modes of chemical etching and / or mechanical penetration by the bioeroders. The importance of the periostracum as protective layer was demonstrated in different species including mussels [36–37] and the black pearl oyster [38]. [36] and [37] report *Perna perna* (Mollusca: Bivalvia) from South Africa that were bioeroded by phototrophic endoliths. Traces of microbioerosion were commonly absent in small *P*. *perna* and present only in areas where the periostracum had been abraded [36–37]. All of these substrates are benthic whereas *Nautilus* actively swims in the water column. Even though the water column is not *per se* a barrier for bioeroder larvae, the rate of successful colonization might partly be limited compared to shells littering the seafloor in closer contact to established bioeroder communities. However, this factor is unlikely to explain the complete absence of microbial bioerosion. In contrast to the live catches reported herein, conchs of dead *Nautilus* sampled in shallow and deep water deposits in the same area [39] and those found in a New Caledonian cenote on Lifou [40] indeed show a diverse suite of microbial bioerosion traces. Since this is the case, we can conclude that the pelagic live habit and, most importantly, the periostracum protected the shell during lifetime against microbial bioerosion. In turn, this indicates that the foraminiferans are among those organisms capable of colonizing the pelagic conchs and effectively penetrating the periostracum as well as bioeroding the calcareous shell below. The ability of foraminiferans to overcome organic tissue and shell layers when settling on and bioeroding calcareous skeletons is well known in the case of parasitic species, particularly *Hyrrokkin sarcophaga*, a rosaliniid foraminiferan, [25, 27, 41–42], which is the most prominent example. This foraminifer is dominantly found on the large bivalve *Acesta excavata* (bearing a periostracum) [25]. The study of abandoned attachment scars revealed the presence of secondary bioerosion (e.g., *Saccomorpha* isp., *Entobia* isp., *Orthogonum* isp.) [25] which supports the idea that if the periostracum is removed other organisms are able to penetrate these parts of the shell.

*H*. *sarcophaga* produces the trace *Kardopomorphos polydioryx* [25], an attachment scar reminiscent to morphotype 1 but unlike the latter it penetrates deeper by forming whip-shaped protrusions that eventually completely penetrate the shell, allowing feeding upon the hosts’ soft body [32–33]. This raises the question whether *Rosalina bradyi* or the producers of any of the other three morphotypes followed a parasitic mode of symbiosis as well. However, we rule out this possibility because none of the documented traces in our study completely penetrates the shell, nor are there any signs of host reaction such as defensive precipitation or repair structures of shell material building up blisters or swellings on the inside of the conch, as is typically found in case of bivalves reacting to foraminiferan parasitism [25]. The only 'passive defense mechanism' is overgrowth of the foraminiferans by the black layer—a layer of melanin-rich organic material that is generated on the previous whorl; the mantle is attached to this black layer and precipitates the nacreous shell on top of it [43–44]—and subsequent shell growth during ontogeny. Therefore we suggest commensalism [45] as primary strategy of attachment using *Nautilus* hosts as suitable mobile substrate offering effective protection from predators. In case of the 'naked' foraminiferans producing morphotype 3 and morphotype 4, an additional benefit could be that the organic compounds in the *Nautilus* periostracum and / or shell matrices served as a nutrient source.

The fact that morphotype 3 and morphotype 4 have, to the best of our knowledge, not been previously reported from any other host or substrate, provokes the question, whether the proposed 'naked' foraminiferans that produced these traces might be substrate-specific for *Nautilus*. Morphotype 3 occurs in both shells, the one from the Vanuatu specimen and the shells from New Caledonia. Morphotype 4 was present in the shells from the New Caledonia specimens only. [24] came to the conclusion that foraminiferans could in fact be highly selective bioeroders, and the aforementioned parasitic *H*. *sarcophaga* is an excellent example, since it may be found on various hosts but shows a clear preference for the bivalve *A*. *excavata* (Mollusca: Bivalvia) (and the associated cold-water coral *L*. *pertusa*) and can therefore be interpreted as substrate-specific. The hypothesis that substrate-specificity could hold true also for the present foraminiferan traces on and in *Nautilus* is currently based on negative evidence only, and further micro-bioerosion studies tackling other calcareous substrates from the tropical Pacific or other areas will tell whether these traces are also present in other substrates. Studies of fossil nautiloids in turn could provide fossil holotype material that would allow proper ichnotaxonomical establishment of morphotypes 1 to 4 and could shed light on the evolutionary pathway of this nautilid / foraminiferan association.

## Conclusions

▪ *Syn-vivo* bioerosion in and on the studied shells of *Nautilus* from New Caledonia and Vanuatu is exclusively undertaken by foraminiferans capable of colonizing the pelagic conchs and penetrating the protective nautilid periostracum.
▪ The bioerosion traces morphotype 1 and 2 are attachment scars produced by epilithic rotaliid foraminiferans, while morphotype 3 and 4 are endolithic traces presumably produced by 'naked' foraminiferans.
▪ None of the traces completely penetrated the shell and defensive host reactions were not observed, characterizing the symbiotic relationship as commensalism with the foraminiferans taking advantage of a mobile habitat providing some protection from predators. The organic matrix in the shell of *Nautilus* may have served as nutrient source.
▪ Morphotypes 3 and 4 are potentially substrate-specific for *Nautilus*.
